# Estimating helminth burdens using sibship reconstruction

**DOI:** 10.1186/s13071-019-3687-1

**Published:** 2019-09-16

**Authors:** M. Inês Neves, Joanne P. Webster, Martin Walker

**Affiliations:** 1 Department of Pathobiology and Population Sciences, Royal Veterinary College, University of London, Hawkshead Lane, Hatfield, UK; 2 London Centre for Neglected Tropical Disease Research, London, UK

**Keywords:** Parentage analysis, Sibship reconstruction, Worm burden, Schistosomiasis, Neglected tropical diseases

## Abstract

**Background:**

Sibship reconstruction is a form of parentage analysis that can be used to identify the number of helminth parental genotypes infecting individual hosts using genetic data on only their offspring. This has the potential to be used for estimating individual worm burdens when adult parasites are otherwise inaccessible, the case for many of the most globally important human helminthiases and neglected tropical diseases. Yet methods of inferring worm burdens from sibship reconstruction data on numbers of unique parental genotypes are lacking, limiting the method’s scope of application.

**Results:**

We developed a novel statistical method for estimating female worm burdens from data on the number of unique female parental genotypes derived from sibship reconstruction. We illustrate the approach using genotypic data on *Schistosoma mansoni* (miracidial) offspring collected from schoolchildren in Tanzania. We show how the bias and precision of worm burden estimates critically depends on the number of sampled offspring and we discuss strategies for obtaining sufficient sample sizes and for incorporating judiciously formulated prior information to improve the accuracy of estimates.

**Conclusions:**

This work provides a novel approach for estimating individual-level worm burdens using genetic data on helminth offspring. This represents a step towards a wider scope of application of parentage analysis techniques. We discuss how the method could be used to assist in the interpretation of monitoring and evaluation data collected during mass drug administration programmes targeting human helminthiases and to help resolve outstanding questions on key population biological processes that govern the transmission dynamics of these neglected tropical diseases.

## Background

Human helminthiases are caused by persistent parasitic infections associated with chronic poor health, morbidity and mortality. Many of the most globally prevalent and disabling helminth infections [1] are included in the World Health Organization (WHO) list of neglected tropical diseases (NTDs). These include the soil-transmitted helminthiases, ascariasis, hookworm and trichuriasis; the filariases, lymphatic filariasis and onchocerciasis, and the trematodiases, including the food-borne trematodiases and schistosomiasis [2–4]. They affect at least one billion people worldwide, predominantly the poorest of the poor [5]. The severity of helminthiases and the contribution to transmission of an infected individual is, at least in part, related to the number of parasites within an individual host at a particular time, the so-called worm burden [6, 7].

In many dioecious helminths, adult parasites live within their definitive host during their entire lifespan and fertile female parasites produce eggs or larvae (transmission stages) that are either released directly into the external environment (e.g. soil-transmitted helminth and trematode infections) or migrate to tissues where they can infect insect vectors (e.g. filarial infections). In humans, it is generally impossible to measure worm burdens directly in natural endemic communities (except for chemoexpulsion techniques for soil-transmitted helminths, see for examples [8–11] and more recently [12]) because adult parasites reside in inaccessible locations. Therefore, it is generally only feasible to perform indirect inference, by counting eggs or larvae. Yet this approach is hampered by high variability in counts made from a single individual (both from repeated samples taken at a single time and among samples taken over a short period of time, see for examples [9, 13–16]) and by potential density-dependent constraints on parasite fecundity that can render the worm-egg/larva relationship highly non-linear [6, 7] and in some cases geographically variable [17].

Parentage analysis using molecular data is used widely in molecular ecology [18–20]. Sibship reconstruction is a category of parentage analysis which can be used to estimate the number of parents when genetic data are available on offspring only [21–24]. Essentially, data on neutral genetic markers are used to divide offspring into groups of full siblings (monogamous mating) or groups of full siblings and half siblings (polygamous mating) to reconstruct and identify unique (male and/or female) parental genotypes. Hence the technique has potential to be used as a method of estimating worm burdens ([25], and see for examples [26–28]). We note that estimates of unique parental genotypes (worm burdens) can be further used in combination with estimates of the frequency of full and half siblings (also from sibship reconstruction) to estimate the effective population size (*N*_*e*_) [29–31] and the effective number of breeders (*N*_*b*_) [24] (using a random sample of individuals from a single cohort in a population with overlapping generations [30]). The (uncertain) relationship between *N*_*e*_ and census population size is discussed in detail in Palstra & Fraser [32].

Sibship reconstruction techniques depend fundamentally on the rules of Mendelian inheritance and frequently on the assumption that parental genotypes comprising a host’s worm burden exist at Hardy-Weinberg equilibrium [18]. Inbreeding, population bottlenecks and linkage disequilibrium of neutral markers proximate to genes under natural selection often violate these assumptions and are well discussed in the literature [33, 34], including parentage methods that relax the assumption of Hardy-Weinberg equilibrium and account for inbreeding [34–36]. But statistical questions that arise when using sibship reconstruction to infer the number of fecund adults have received little attention. While it is intuitive that in dioecious species, the number of unique female parental genotypes identified by sibship reconstruction will be limited (to a maximum) by the number of sampled offspring (eggs/larvae, i.e. each offspring can have only a single mother)—and consequently that the number of full sibling families is a minimum estimate of the female worm burden [24]—the statistical properties of how the number of unique parental genotypes in individual hosts relates to the underlying true number of fecund female adults (female worm burden) has not been explored. A clearer exposition of this statistical relationship is therefore essential to interpret accurately how parental genotypes inferred by sibship reconstruction relate to worm burdens.

Here, we develop a statistical approach to estimate the number and associated uncertainty of fecund female worms from data on the number of unique female parental genotypes identified by sibship reconstruction. We illustrate the technique using data from a recent study [28] where sibship reconstruction was used to identify unique parental genotypes of female *Schistosoma mansoni* infecting schoolchildren in Tanzania. We discuss potential applications in the context of monitoring and evaluation of mass drug administration programmes and resolving outstanding questions on the fundamental population biology of human helminthiases.

## Methods

We define *N* as the number of fecund female schistosomes in a host. We also refer to *N* as the (female) worm burden. We assume that the *N* worms in a host are genetically unrelated and therefore each has a unique genotype of neutral genetic markers (e.g. microsatellites [28]). We define *m* as the number of miracidal offspring (hatched from eggs) sampled from a host and *n* as the number of unique female parental genotypes identified by sibship reconstruction [20, 21]. We further assume that the pool of eggs (hatched to miracidia) to sample from is large compared to *N* and that female worms within a host are equally fecund (i.e. each worm makes an equal contribution to the population of offspring). Under these assumptions, the probability of identifying *n* female parental genotypes from a sample of *m* miracidia is described by the unique items distribution [37],

1 $$f\left( {n |N,m} \right) = \frac{{(N)_{n} }}{{N^{m} }}\left\{ {\begin{array}{*{20}c} m \\ n \\ \end{array} } \right\},$$

where *(N)*_*n*_ is the falling factorial,

2 $$(N)_{n} = \frac{N!}{{\left( {N - n} \right)!}}, \quad {\text{for }}n \le N$$

and $$\left\{ {\begin{array}{*{20}c} m \\ n \\ \end{array} } \right\}$$ is a Stirling number of the second kind.

The expected value of *n* is given by [37],

3 $$E\left( n \right) = \frac{{N^{m} - (N - 1)^{m} }}{{{\text{N}}\left( {m - 1} \right)}}$$

such that the bias in *E(n)* as an underestimate of *N* expressed as a percentage is *[E(n)−N]/N*.

Inference on *N* from *n* identified parental genotypes is derived from the posterior probability *f(N|n,m)* using Bayes’ theorem,

4 $$f(N|n) \propto f(n|N)f\left( N \right),$$

where *m* is omitted for brevity and *f(N)* denotes the prior probability of a host having a worm burden *N* (i.e. the prior probability of *N* fecund female worms). We sampled from the posterior distribution *f(N|n)* using the following sampling importance re-sampling algorithm [38, 39]:

i. draw a random sample of *N* from an integer uniform distribution *g(N|n)* for *N ∈ [n, N*_*max*_*]*
ii. calculate weights associated with each value of *N*, given by *w(N)* = *f(n|N)f(N)*
iii. re-sample *N* with replacement using the weights calculated in step 2

We set *N*_*max*_ (the maximum conceivable number of fecund female worms) to 350 in accordance with the autopsy observations made by Cheever [40]. This required the factorial in Equation 1 to be evaluated using Stirling’s approximation for *N* > 170,

5 $$\ln N! = N { \ln }N - N$$

We illustrate our statistical approach using genotypic data on schistosomes from schoolchildren in Tanzania collected in 2005, 2006 and 2010 [28]. In this case, *n* corresponds to the inferred number of unique female *S. mansoni* genotypes within each individual child (estimated by sibship reconstruction using multiplexed microsatellite genotypic data), and *m* corresponds to the number of sampled miracidia per child (ranging from 1 to 20). The number of fecund female worms *N* was estimated using a weakly informative (uniform) and informative negative binomial priors (*W* = 45 and *k* = 0.5 or *k* = 1). The analysis was performed in R [41] version 3.5.1.

## Results

### Relationship between number of unique parental genotypes and worm burden

**Fig. 1:**
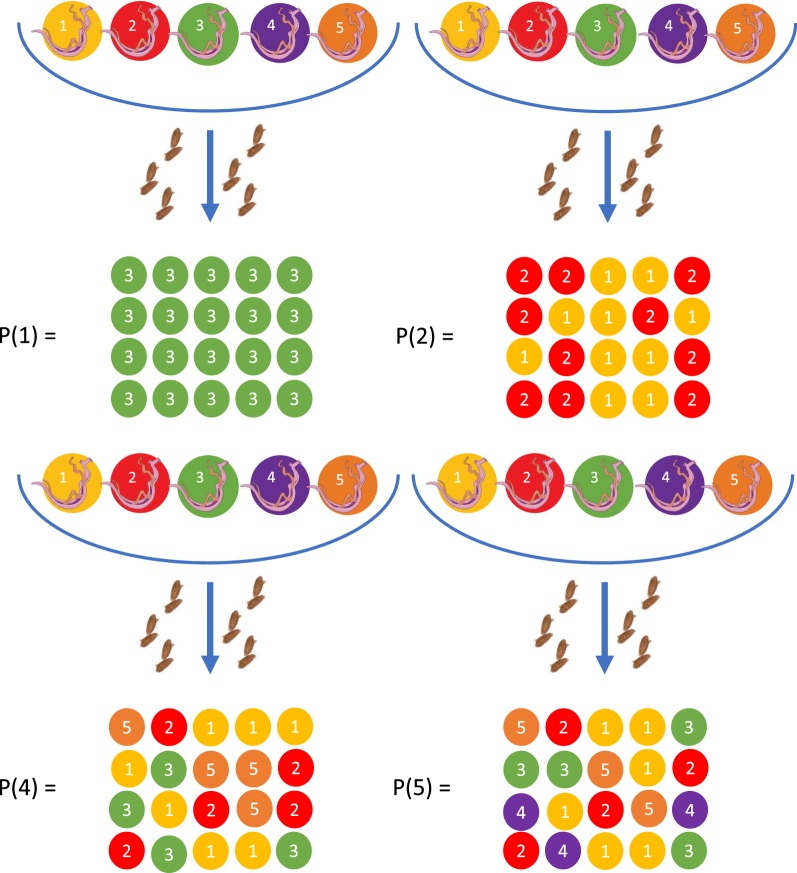
Schematic representation of the unique items distribution. If a host is infected with five female worms/parental genotypes *N* = 5, and *m* = 20 miracidia/offspring are sampled, genotyped and subjected to sibship analysis (assuming 100% accuracy of the sibship reconstruction) then P(1), P(2), P(4) and P(5) are the probabilities of identifying exactly *n* = 1, *n* = 2, *n* = 4 and *n* = 5 unique parental genotypes. The expected value of *n*, *E(n)*, depends on the values of *N* and *m* following the expression given in Equation 3. The bias in *E(n)* as an (under)estimate of *N* (expressed as a percentage) is *[E(n) − N]/N*. This figure was created using Servier Medical Art according to a Creative Commons Attribution 3.0 Unported License guidelines 3.0 (https://creativecommons.org/licenses/by/3.0/)

**Fig. 2:**
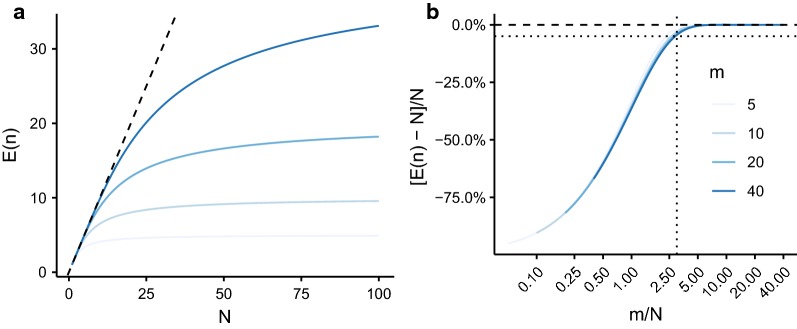
Expected value and bias in the identified number of parental genotypes when the true number of parental genotypes is known. The identified number of parental genotypes by sibship reconstruction is denoted *n* with expected value *E(n)* derived from the unique items distribution. The true number of parental genotypes (the fecund female worm burden) is denoted *N* and the number of sampled miracidial offspring *m*, increasing from 5 to 40 per host, from light to dark blue. The dashed line in panel **a** corresponds to the relationship *E(n)* = *N*, indicating an unbiased estimate of *N*. In panel **b** the bias is expressed as a percentage underestimate of *N*, *[E(n) − N]/N* which is plotted against the ratio *m/N*. The horizontal dashed line in panel **b** indicates a 5% underestimate; the vertical dashed line is plotted at *m/N* = 3, the approximate ratio above which *n* is an underestimate of less than 5%

Intuitively, the observed number *n* of unique female parental genotypes is a biased underestimate of the true number of female parental genotypes *N* present within a host, here defined as the fecund female worm burden. Clearly, *n* cannot be greater than the number of sampled (miracidial) offspring *m* [24] (i.e. when no full or half sibling pairs are identified from a sample of *m* offspring each of the *m* offspring has a different mother). The degree of bias can be quantified using the properties of the unique items distribution [37] (Fig. 1) under the assumptions that the number of offspring is large compared to *N* and that female worms within a host are equally fecund (i.e. there is an equal probability of sampling offspring from any female, see Methods). The relationship between the expected number (mean) of unique female parental genotypes *E(n)*, the number of sampled offspring *m* and the fecund female worm burden *N* is shown in Fig. 2. The bias in *n* as an estimate of *N* is strongly dependent on the ratio *m*/*N*, such that to achieve less than a 5% underestimate of *N* one typically requires a sample of at least three times as many offspring as fecund female worms (Fig. 2b).

### Estimating worm burdens

**Fig. 3:**
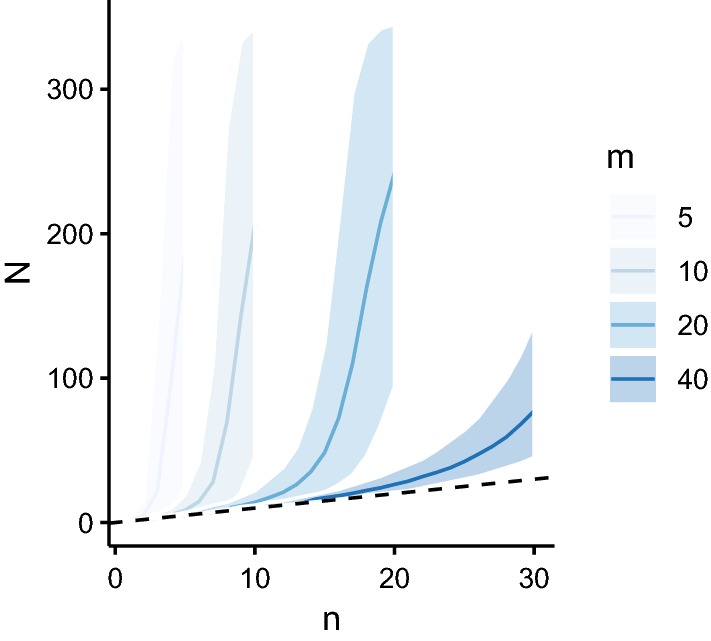
Expected mean value and 95% confidence intervals of the estimated number of fecund female worms for increasing number of parental genotypes identified by sibship reconstruction. The number of fecund female worms is denoted *N* and is plotted against the number of female parental genotypes *n* identified by sibship reconstruction. The line corresponds to the expected value (mean) *E(N)* of the estimated number of fecund female worms and the shaded bands denote 95% confidence intervals (CIs). *E(N)* and 95% CIs are derived from the posterior distribution of *N*, given *n* and the number of sampled miracidial offspring *m*, increasing from 5 to 40 per host, from light to dark blue. The dashed line corresponds to the relationship *E(N)* = *n*

**Fig. 4:**
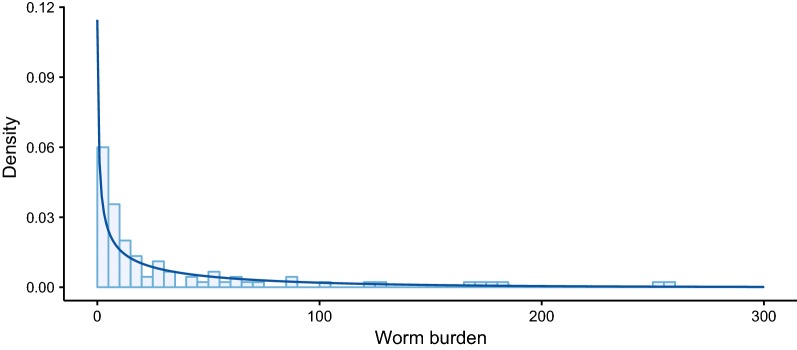
Data on the number of adult female *Schistosoma mansoni* extracted at autopsy from 96 individuals and fitted negative binomial distribution. Of the 103 cases described in the autopsy study [40], only 96 were used in the analysis. One was excluded for lack of female worm counts, 5 cases were excluded due to treatment with antimonials before investigation, and one case was excluded for being an extreme case of schistosomal colitis, with 1608 worm pairs. The negative binomial distribution was fitted by maximum likelihood, estimating the mean *W* = 45.51 and overdispersion parameter *k* = 0.47

The purpose of sibship reconstruction in this context is to estimate a host’s female worm burden when it is unknown (i.e. *N* is unknown). We make inference on *N* and associated uncertainty from its posterior distribution, given an observed number of unique parental genotypes *n* and sampled (miracidial) offspring *m* (see Methods for details). The expected value (mean) *E(N)* and 95% confidence intervals are depicted in Fig. 3 for increasing numbers of identified unique female parental genotypes *n* and sampled (miracidial) offspring *m*. This relationship is based on the mild (weakly informative) *a priori* belief that hosts cannot harbour more than 350 female worms, but are otherwise equally likely to harbour any number between 1 and 350 worms. This maximum was chosen based on an autopsy study [40] that counted adult female *S. mansoni* directly from 103 people (Fig. 4). Without this constraint, the upper confidence intervals in Fig. 3 as *n* → *m* would be unbounded, tending to infinity.

**Fig. 5:**
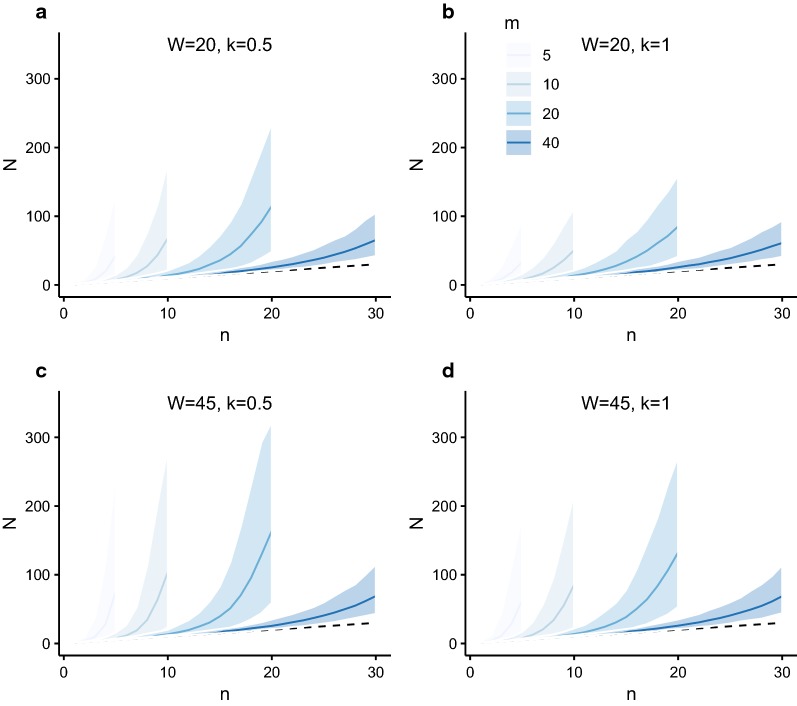
Expected value and 95% confidence intervals of the estimated number of fecund female worms for increasing number of female parental genotypes identified by sibship reconstruction using informative prior distributions. The posterior distribution of the number of fecund female worms is denoted *N* and is plotted against the number of female parental genotypes *n* identified by sibship reconstruction. The line corresponds to the expected value (mean) *E(N)* and the shaded bands denote 95% confidence intervals (CIs). *E(N)* and 95% CIs are derived from the posterior distribution of *N*, given *n* and an informative negative binomial prior distribution for *N*. The mean *W* and overdispersion parameter *k* are: *W* = 20 and *k* = 0.5 in panel **a**; *W* = 20 and *k* = 1 in panel **b**; *W* = 45 and *k* = 0.5 in panel **c**, and *W* = 45 and *k* = 1 in panel **d**. In each panel the dashed line corresponds to the relationship *E(N)* = *n*. The number of sampled miracidial offspring *m*, increases from 5 to 40 per host, from light to dark blue

The autopsy data shown in Fig. 4 follow a pattern of overdispersion that is typical of helminths and are well described by a negative binomial distribution. Hence, when estimating *N* in a population, it may be reasonable to assume that a randomly selected individual is more likely to have a low than a high worm burden. We incorporated this *a priori* belief using an informative negative binomial prior distribution, parameterised with a mean *W* and overdispersion parameter *k*, exploring the influence of *W* and *k* on estimates of *N* (Fig. 5). The values *W* = 45 and *W* = 20 chosen for this analysis were informed by the autopsy data; the mean *W* = 45 was estimated by fitting a negative binomial distribution to the data and the mean *W* = 20 was chosen because the autopsies were undertaken on the bodies of patients who had been terminally ill, many of whom had suffered severe schistosomiasis. Thus, we considered that the sample (with a mean *W* = 45) was likely biased towards heavier schistosome infections. The values of *k* = 0.5 and *k* = 1 were chosen as plausible considering both the autopsy data (Fig. 4) and values typically observed for other helminthiases [6, 42]. It is clear from Fig. 5 that higher values of *W* result in higher estimated values of *N* and associated degrees of uncertainty (compare Fig. 5c, d with Fig. 5a, b). Higher assumed (*a priori*) aggregation (lower *k*) results similarly in higher estimated values of *N* and associated degrees of uncertainty (compare Fig. 5a, c with Fig. 5b, d).

### Illustration using genotypic data on schistosomes from schoolchildren in Tanzania

Gower et al. [28] used multiplexed microsatellite genotypic data of miracidia (hatched from eggs) sampled from 151 schoolchildren in Tanzania to identify by sibship reconstruction unique female *S. mansoni* genotypes within each individual child. Full-pedigree likelihood methods were used to infer sibship using the COLONY software package [43] and the number of miracidia sampled per child ranged from 1 to 20. Samples were collected in 2005, 6 months before the start of mass drug administration with praziquantel, in 2006 and in 2010.

**Fig. 6:**
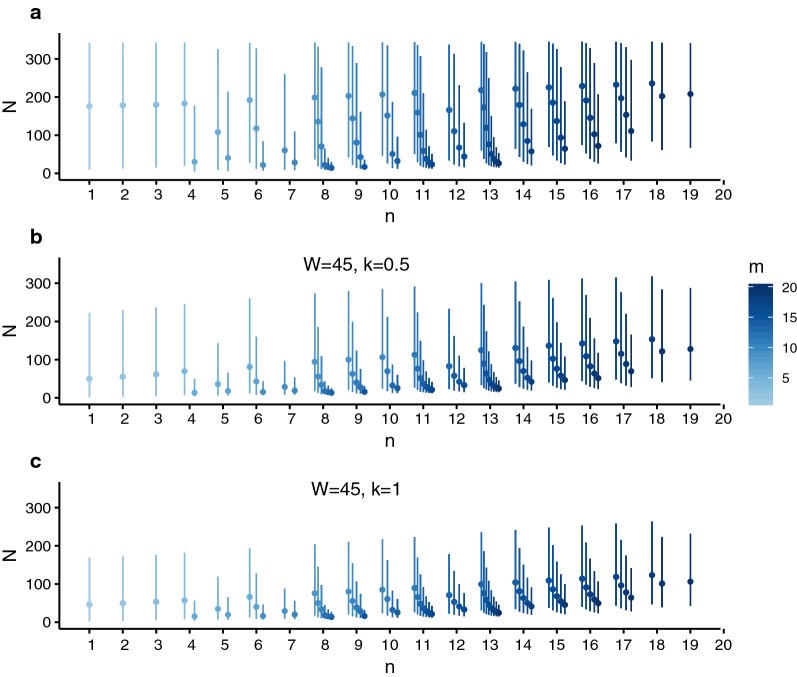
Estimated number of fecund female worms using genotypic data on *Schistosoma mansoni* miracidia collected from schoolchildren in Tanzania. The technique is illustrated using data from a recent study [28], where sibship reconstruction was used on multiplexed microsatellite genotypic data of miracidia collected from 151 schoolchildren in Tanzania, to identify the number of unique parental genotypes of *S. mansoni* in each individual. The posterior distribution of the number of fecund female worms *N* was estimated for each of 150 schoolchildren, given the identified number of unique parental genotypes *n*, the number of sampled miracidial offspring *m*, and using either a weakly informative (uniform) prior distribution or an informative negative binomial prior, the latter parameterised by the mean number of female worms per host *W* and overdispersion parameter *k*. The weakly informative prior in panel **a** is described by a uniform distribution ranging from 1 to 350 (female worms). The informative negative binomial prior in panel **b** is defined by *W* = 45 and *k* = 0.5, and in **(c)** by *W* = 45 and *k* = 1. The negative binomial prior parameterisations were informed by the autopsy data from Cheever [40]. The number of sampled miracidial offspring *m*, increases from 1 to 20 per host, from light to dark blue

The estimated number of fecund female worms *N* from each child in 2005, 2006 and 2010 are shown in Fig. 6, using the weakly informative (uniform) and informative negative binomial priors (*W* = 45 and *k* = 0.5 or *k* = 1). The results show that the number of unique female genotypes *n* for each child was substantially lower than the corresponding estimated posterior number of fecund female worms, *N*. For identical values of *n*, the posterior mean *E(N)* and associated uncertainty decreases as the number of miracidia *m* increases. As *n* becomes close to *m*, the estimated posterior of *N* becomes dominated by the prior distribution.

The mean and degree of uncertainty of the posterior estimates of *N* were considerably greater using a weakly informative prior (Fig. 6a, i.e. using a uniform prior that individuals are equally likely to harbour any number of worms between 1 and 350), compared to those using informative negative binomial prior distributions. The particular parameterisation of the negative binomial prior results in different posterior distributions of *N* as illustrated using *W* = 45 and *k* = 0.5 (Fig. 6b) or *W* = 45 and *k* = 1 (Fig. 6c).

## Discussion

Parentage analysis by sibship reconstruction can be used to identify unique parental genotypes from genetic information on helminth offspring, which are typically more accessible than adult parasites, particularly for human helminthiases. We have developed a novel statistical approach to estimate—with associated measures of uncertainty—the number of fecund adult helminths from the number of unique parental genotypes identified by sibship reconstruction. We have illustrated the approach using genotypic data on *S. mansoni* miracidia collected from schoolchildren in Tanzania, highlighting the critical importance of (miracidial) offspring sample sizes to the precision of female worm burden estimates. This work provides a statistical exposition of using parentage analysis to estimate helminth worm burdens and thus a step towards a more robust application of this technique.

The potential of sibship reconstruction approaches to estimate the number of fecund adult helminths infecting a host when direct counts are impossible has been recognised for at least 15 years [25], but has only been applied relatively recently [24, 26–28] (see also [44]). Hitherto, the majority of studies have only used sibship reconstruction to identify the number of unique (fecund) parental genotypes [26–28] or analogously the number of full-sibling families (indicating the minimum number of worm pairs present within a host) [24], but have rarely attempted to relate these results to the underlying number of fecund female worms. The approach outlined in this paper provides a method to quantify the uncertainty (with credible intervals) of these estimates and has a number of potential population biological and epidemiological applications.

Many of the human helminthiases on the WHO list of NTDs are targeted for control or elimination, principally by mass drug administration (MDA) of anthelmintics to affected communities [45]. Epidemiological studies and routine monitoring and evaluation activities to assess the impact of MDA typically rely on egg counts or other indirect measures of infection intensity (such as detection of eggs/larvae by more sensitive molecular methods, e.g. [12, 46]). But the interpretation of such data can be complicated by uncertain and non-linear [6, 7] or geographically variable [17] relationships with worm burden. Estimates of worm burden could therefore provide a useful approach to complement and aid the interpretation of such data. For example, in populations where egg or larva counts are higher than expected after years of MDA (in schistosomiasis these are referred to as ‘hot-spots’, see for example [47, 48]), worm burden estimates could be used to distinguish programmatic deficiencies (e.g. poor coverage or missed MDA rounds) associated with higher than expected worm burdens from population biological processes associated with as-expected low worm burdens but higher than expected egg/larva counts (as occurs from the relaxation of density-dependent fecundity as worm burdens are reduced [49]).

The importance of density-dependent fecundity in interpreting egg count data is particularly pertinent to schistosomiasis because it remains unclear whether this fundamental population biological process operates in either of the two most globally important species, *S. mansoni* and *S. haematobium* [40, 50–53]. The use of sibship reconstruction to estimate worm burdens could help resolve this long-running debate by exploring the association between egg counts and inferred female worm burdens, albeit using robust statistical methodologies to account for the likely substantial degree of measurement error (uncertainty) associated with the covariate (worm burden) estimate ([54] and see for example [55]). Moreover, the approach could also be used to revisit the relationship between worm burden and the relatively new antigen-based diagnostics for schistosomiasis, inference on which has been hitherto restricted to comparison with egg counts (see [56] and references therein).

The sample size of (miracidial) offspring is, however, a key limitation to the precision with which worm burdens can be estimated. Defining adequate sample sizes to determine population-level genetic diversity of human helminthiases has received attention, and in particular for schistosomiasis [57, 58]. But the sampling strategy required to make individual-level parentage inference is a different proposition. Clearly, the more parasite offspring that can be collected and genotyped, the more robust the inference on worm burdens and thus ideally one would collect and genotype as many offspring (eggs/larva) as possible. This is feasible for soil-transmitted helminthiases and trematodiases, including schistosomiasis, by whole stool sampling [59, 60] or by using much greater quantities of stool than are used for routine diagnosis (e.g. [61]). This is important both in lightly infected individuals, where routine methods for counting eggs may frequently find no infection due to poor sensitivity [62–65], and in heavily infected individuals where sampling effort may otherwise be compromised by the greater ease of collecting parasite material from smaller quantities of stool.

One potential sampling approach would be to sample offspring proportionally to parasitological or other molecular indicators of the intensity of infection per individual. Although indirect measures of quantification are unreliable indicators of worm burden [9, 12, 65], which indeed is a key premise of using parentage analysis to estimate worm burdens, they provide some, albeit noisy, information at an individual level with which to motivate desired sample sizes. For example, one could chose a minimum baseline offspring sample size of 10 (which would be sufficient to estimate with reasonable precision female worm burdens of 3–4) and increase this value in proportion to the percentile of observed intensity indicators (such as eggs per gram of faeces per individual) obtained from a group or population of individuals.

The choice of prior distribution is a further important consideration when estimating worm burdens from the results of sibship reconstruction. The most cautious approach is to assume that any number of worms is equally as likely, and indeed that there is no upper limit on how many worms a host may harbour (here 350 female worms was considered a maximum for *S. mansoni* based on the human autopsy data [40], Fig. 4, but also to bound the posterior distribution of female worm burden as *n* → *m*). This will inevitably lead to the greatest uncertainty in worm burden estimates (Fig. 6). Using a negative binomial prior is well justified based on the wealth of empirical evidence from a variety of human and animal helminth infections [6, 42]. The difficulty arises with parameterising this prior distribution; both the mean and the degree of overdispersion will undoubtedly vary considerably among settings and particular contexts. The best approach is likely to assemble estimates made under different plausible prior assumptions. But it is also important to note that the influence of the prior is most pronounced in cases where the number of identified parental genotypes approaches the number of sampled offspring. Therefore, in the majority of hosts (with low worm burdens), the choice of prior may have limited substantive impact if relatively large offspring sample sizes are achieved.

Aside from the statistical properties of the relationship between parental genotypes, the number of sampled offspring and the underlying (female) worm burden, there exist a variety of population biological and genetic assumptions not considered here explicitly that may affect the accuracy of sibship reconstruction [20]. Methods for identifying unique parental genotypes from offspring genetic data are probabilistic [21] and thus will have some inherent uncertainty. Accurate inference particularly depends on the assumption of either a monogamous or polygamous mating system. Parental genotypes were identified from the Tanzanian data under the common assumption that schistosomes are strictly monogamous [6, 66] (as opposed to the ubiquitous assumption of polygamy among other human helminthiases [6]) although in model systems mate changes and polygamy do occur [67]. Studies have also shown that mating competition occurs between different species (and even different genotypes), and increased polygamy exists in high selective pressure environments [68, 69]. The assumption of a strictly monogamous mating system implies that the number of identified female genotypes is equal to the number of male genotypes, and therefore, only full-sibs sharing the same mother and father can be present in the sampled offspring. Under the assumption of a polygamous mating system, half-sib pairs sharing the same mother but different fathers can exist. Therefore, if strict monogamy does not hold, half-sib pairs may be erroneously missed, potentially overestimating the number of unique parental genotypes because of a failure to infer sibling relationships among the sampled offspring. Notwithstanding, a recent empirical comparison of the results of sibship reconstruction under the assumption of either a monogamous or a polygamous schistosome mating system found the assumption to have relatively little impact [24].

An important statistical limitation of this work is the assumption of equally fecund female worms within a host, i.e. that each worm makes an equal contribution to the population of offspring. A previous study used sibship reconstruction to quantify family structure in miracidial offspring (i.e. numbers of miracidia per full-sib family) and indicated that the reproductive success of breeding schistosomes was likely skewed, resulting in differential representation of each family in the offspring pool [24]. The probability of identifying exactly *n* female parental genotypes from a sample of *m* miracidia will be affected by this unequal contribution as in turn will the estimated number of *N* parental genotypes. Changing this assumption to account for variable reproductive output will be explored in future studies.

The assumptions of Mendelian inheritance and Hardy-Weinberg equilibrium of parental genotypes [18] are also limitations of parentage analysis techniques (but see [34–36]) for methods that relax the assumption of Hardy-Weinberg equilibrium). These assumptions may be violated by many factors, particularly during MDA programmes [70–72]. Even before MDA, assumptions of random mixing may not be upheld if genetically related parasites are transmitted together in so-called ‘clumps’ or ‘packets’, as described for directly-transmitted helminth infections [73–75]. This process, alongside other host and genotype-dependent immunity factors [76], would promote inbreeding [77] and departure from Hardy-Weinberg equilibrium. For schistosomiasis, although asexual reproduction within intermediate snail hosts may also seemingly enhance inbreeding within the definitive (human) hosts, the mixing in water bodies of free-living cercariae released from snails will likely act as a counteractive balance.

In practice, simulation studies have shown that inbreeding or relatedness among male and female parents has only a small effect on the accuracy of sibship reconstruction [36]. Therefore, for dioecious species (like schistosomes) it has been recommended that sibship reconstruction is performed under the assumptions of Hardy-Weinberg equilibrium (no inbreeding), except when there is strong evidence to the contrary and the level of inbreeding is high [78]. Moreover, the statistical relationship between the estimated number of fecund female parasites and the number of unique parental genotypes identified from a finite sample of (miracidial) offspring will be unaffected by the specific assumptions used for sibship reconstruction. Notwithstanding, if inbreeding is suspected as being high, sibship reconstruction can be implemented with or without assumptions of Hardy-Weinberg equilibrium to see how the identified number of unique parental genotypes may differ. The statistical approach presented here could be applied to different numbers of identified genotypes to estimate the corresponding number (and associated uncertainty) of fecund female worms as a form of sensitivity analysis.

Ultimately, validation of the approach outlined in this work would require that predictions be tested against directly observed adult (female) worm burdens. This is possible for human soil-transmitted helminths because adult worms can be expelled and counted following anthelmintic treatment [8–12]. For schistosomiasis and many other helminthiases, validation in related animal parasites that can be counted by dissection (e.g. *S. bovis* infections in cattle) or laboratory rodent studies [79–81]  is the most amenable option. A drawback of this approach is that the population processes that shape the population genetics, and therefore the validity of the core assumptions inherent to sibship reconstruction, may be different in animal compared to human populations, especially populations under the influence of MDA programmes or frequent treatment.

## Conclusions

An emerging use of sibship reconstruction is to identify the number of unique parental genotypes of human helminth infections from genetic information on their offspring. This is an important application of a well-developed parentage analysis technique because in many human helminthiases it is not possible to access adult worms, while it is relatively easy to sample their offspring. We have developed a statistical method to relate the number of identified parental genotypes to the underlying fecund female worm burden, highlighting the critical importance of offspring sample size on the bias and precision of worm burden estimates. The technique has potential applications in aiding the interpretation of routine monitoring and evaluation data collected during helminth control programmes and could contribute to resolving some outstanding population biological questions, particularly on the operation of density-dependent processes.

## Data Availability

Data supporting the conclusions are included within the article. The code is fully operational under R version 3.5.1 [41] and is freely available for download from https://github.com/minesneves
